# Modelling Hydrologic Processes in the Mekong River Basin Using a Distributed Model Driven by Satellite Precipitation and Rain Gauge Observations

**DOI:** 10.1371/journal.pone.0152229

**Published:** 2016-03-24

**Authors:** Wei Wang, Hui Lu, Dawen Yang, Khem Sothea, Yang Jiao, Bin Gao, Xueting Peng, Zhiguo Pang

**Affiliations:** 1 Ministry of Education Key Laboratory for Earth System Modeling, and Center for Earth System Science, Tsinghua University, Beijing, China; 2 The Joint Center for Global Change Studies, Beijing, China; 3 Department of Hydraulic Engineering, Tsinghua University, Beijing, China; 4 Mekong River Commission Secretariat (MRCS)/OSP, Phnom Penh, Cambodia; 5 School of Water Resources and Environment, China University of Geosciences (Beijing), Beijing, China; 6 Remote Sensing Center, Institute of Water Resources and Hydropower Research, Beijing, China; University California Los Angeles, UNITED STATES

## Abstract

The Mekong River is the most important river in Southeast Asia. It has increasingly suffered from water-related problems due to economic development, population growth and climate change in the surrounding areas. In this study, we built a distributed Geomorphology-Based Hydrological Model (GBHM) of the Mekong River using remote sensing data and other publicly available data. Two numerical experiments were conducted using different rainfall data sets as model inputs. The data sets included rain gauge data from the Mekong River Commission (MRC) and remote sensing rainfall data from the Tropic Rainfall Measurement Mission (TRMM 3B42V7). Model calibration and validation were conducted for the two rainfall data sets. Compared to the observed discharge, both the gauge simulation and TRMM simulation performed well during the calibration period (1998–2001). However, the performance of the gauge simulation was worse than that of the TRMM simulation during the validation period (2002–2012). The TRMM simulation is more stable and reliable at different scales. Moreover, the calibration period was changed to 2, 4, and 8 years to test the impact of the calibration period length on the two simulations. The results suggest that longer calibration periods improved the GBHM performance during validation periods. In addition, the TRMM simulation is more stable and less sensitive to the calibration period length than is the gauge simulation. Further analysis reveals that the uneven distribution of rain gauges makes the input rainfall data less representative and more heterogeneous, worsening the simulation performance. Our results indicate that remotely sensed rainfall data may be more suitable for driving distributed hydrologic models, especially in basins with poor data quality or limited gauge availability.

## Introduction

**Fig 1:**
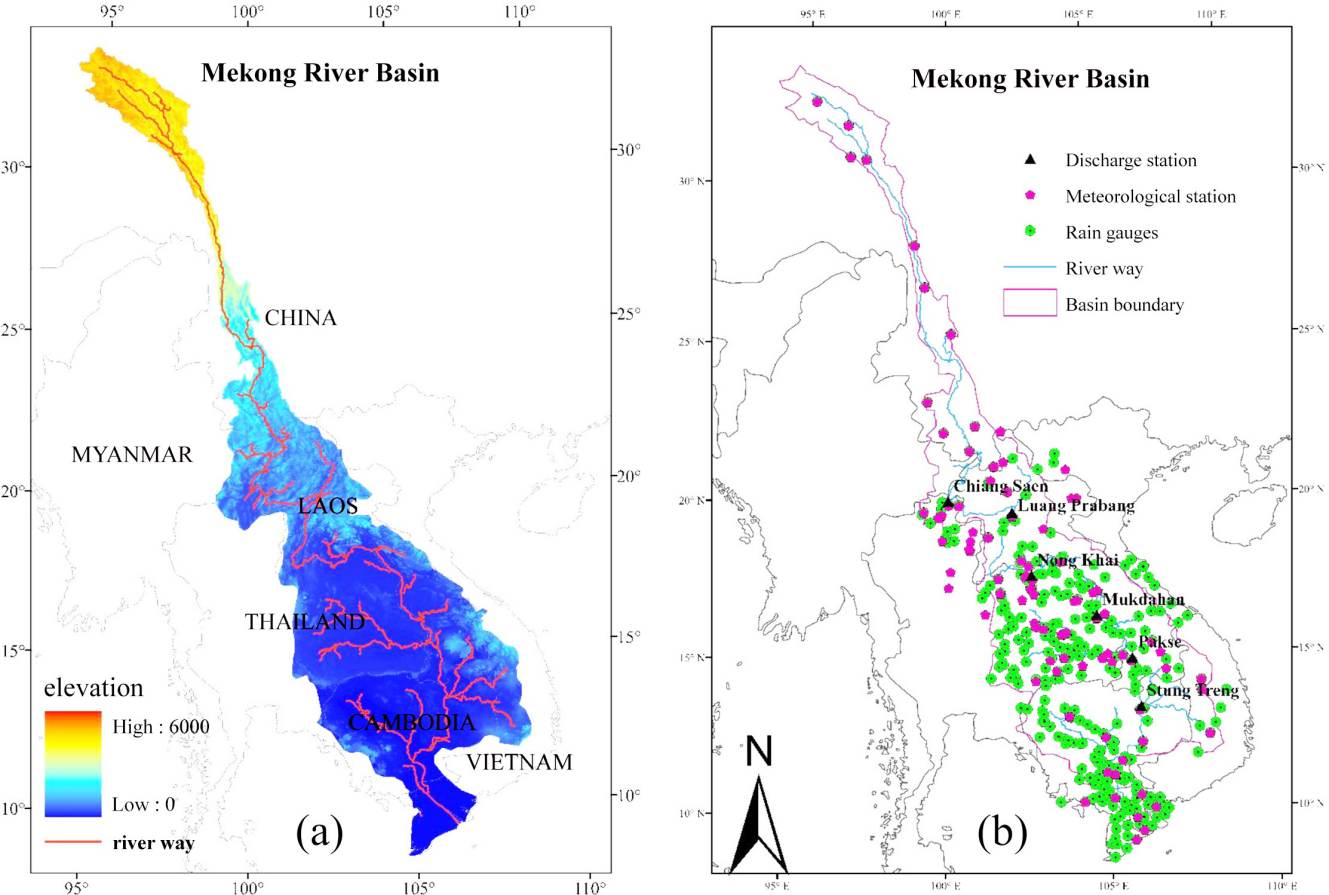
**Mekong River Basin:** (a) the natural basin; (b) the gauge locations used in the simulations.

The Mekong River is the most important trans-boundary river in Southeast Asia. It flows from the Tibetan Plateau in China through China’s Yunnan province, Burma, Laos, Thailand, Cambodia and Vietnam before finally discharging into the South China Sea (Fig 1(A)). It is the tenth largest river in the world, with a length of almost 4,900 km, a total catchment area of 795,000 km^2^and an average discharge of 14,500 m^3^/s [1].

Over the last few decades the Mekong River Basin (MRB) has experienced rapid economic development, urbanization, and population growth. These changes have adversely affected on the environmental and hydrological resources of the basin. Water-related problems such as water shortages and water pollution are worsening in the basin [2]. Moreover, a changing global climate will place additional pressure on the MRB. Research has been conducted to assess the impacts of climate change on freshwater resources within the river basin [3–7]; however, significant uncertainties still exist regarding projections of future water resources [8]. Additionally, the trans-boundary nature of the Mekong adds complexity to the water related problems. This abundant water resource also causes disputes between the countries along the flow path. These types of disputes are common along trans-boundary rivers [9–10].

Numerous scientist have conducted hydrologic simulations in the MRB to mitigate the previously mentioned problems. Kite [11] used a semi-distributed land-use runoff process (SLURP) hydrological model to assess the impacts of basin development on fishery productivity and climate change in the Lower Mekong Basin (LMB) using climatic, topographic and land cover data. A Decision Support Framework (DSF) was used by the International Water Management Institute and Mekong River Commission to apply the Soil and Water Assessment Tool (SWAT) hydrological model, the Integrated Quantity Quality Model (IQQM) and the hydrodynamic ISIS model to project flow changes based on different climate and development scenarios [6]. The Variable Infiltration Capacity (VIC) model was applied to the entire Mekong Basin by Costa-Cabral et al. [12] to address the relative influences of spatial and temporal rainfall and soil moisture variabilities. The analysis focused on hydrological, sediment transport and carbon cycle effects on runoff generation in the MRB. Yang and Katsumi [13] developed a continental scale model that included sub-grid hydrological parameterization based on hill slope scale morphology, soil and land cover use in Asia. These studies have helped shape the policy and planning debate; however, further research is needed. The SLURP and SWAT models are only semi-distributed models and cannot provide detailed distributions of hydrological variables. VIC is designed for large-scale areas and possesses a relatively low spatial resolution. In addition, the performance of these models has been fairly poor, with a relative bias of -9.3% at Pakse [11] and monthly Nash-Sutcliffe coefficients of 0.7 at Mukdahan [13] and 0.72 at Stung Treng [12]. Moreover, projections based on future climate and water resource scenarios should focus on the entire basin rather than just a part, particularly because the Mekong River is a trans-boundary river. At the basin scale, results and strategies can be shared among the countries through which the Mekong River flows.

In addition, the climate in the MRB is extremely complex, with high spatial variability. Different sub-basins often exhibit distinct drainage patterns [1]. The rainfall-runoff relationship varies from the northern Tibetan plateau to the southern edge of the Mekong delta. From this perspective, a simple lumped or semi-distributed model with a low resolution may not accurately represent the complex climate, geomorphology, and land cover characteristics of the MRB. A high spatial resolution distributed hydrological model (DHM) must be developed for the MRB. Such a DHM should encompass the temporal and spatial variabilities of catchment conditions and meteorological inputs, providing an improved representation of the hydrological processes compared to a traditional lumped model.

However, DHMs generally require large amounts of detailed catchment information or input data, such as meteorological inputs and land use, to describe the spatial-temporal variations of the basin [14, 15]. Insufficient or inaccurate data would lead to poor simulation results using a DHM, especially in an underdeveloped area such as the MRB. Because it is a less-developed area and a trans-boundary basin, in situ data are difficult to obtain for the MRB. In addition the data quality cannot be guaranteed. Furthermore, the available rain gauges and meteorological stations are sparsely and unevenly distributed. More are concentrated in cultivated regions along the main river, while few are located in remote mountainous regions. Some researchers note out that gauge data or field survey data can only represent local scale information, which may vary from large-scale data [16]. All of these factors limit the application of a DHM in the MRB.

The development of remote sensing (RS) technology has allowed for the integration of RS data and DHM over the last few decades [17]. Massive amounts of earth observation data can be easily obtained via the internet, providing the possibility for hydrological cycle simulations in areas with poor data availability [18]. Consequently, RS has largely overcome the difficulties associated with obtaining reliable long-term in situ data at a large scale. RS data are widely spatially distributed and readily accessible. Many types of remote sensing data have been applied to hydrology research. Among these, remote sensing-based precipitation is the most popular product [19].

Precipitation is the key forcing variable in hydrological models. The spatial and temporal patterns, intensity and duration of precipitation significantly affect the hydrological cycles [20]. Therefore, various types of rainfall data, including in situ data and RS products, should be tested and comprehensively considered for use in DHMs [21]. Multiple RS-based precipitation products have been applied to hydrological simulations in different basins and on various scales [22, 23]. RS products are well suited for hydrological modeling in both humid areas and arid areas, exhibiting a considerable local bias. However, previous studies have seldom used precipitation data sets as inputs to MRB hydrological models.

This study conducts simulations of the daily hydrological process in the MRB using a Geomorphology-Based Hydrological Model (GBHM) driven by two forcing rainfall data sets: traditional station data and RS grid data. The study aims to assess the feasibility of simulating the hydrological process in a basin with scarce ground-based information using a DHM driven by RS data. The GBHM and data used in this study will be introduced in the next section, followed by the calibration and validation results. A further analysis and comparison of the GBHM driven by the two rainfall data sets is then conducted. Finally, the paper concludes with a discussion and summary.

## Materials and Methods

In this study, two experiments were conducted after a distributed hydrological model was built for the MRB using free public data. In one experiment, the model was driven by gauge-observed rainfall data (gauge simulation), while the other experiment was driven by RS rainfall data (TRMM simulation). The model was calibrated with these two types of input rain data from 1998 to 2001. The performances of the GBHM for the validation period of 2002 to 2012 was compared using several assessment indices. Then, a further comparison between the two simulations was conducted. The comparison included two aspects: (1) changing the length of the calibration period to 2, 4 and 8 years to assess the impact on the two simulations; and (2) comparing the gauge simulation and TRMM simulation for a particular year to explore how an uneven distribution of rainfall gauges impacts the simulated discharge.

### Model description

**Fig 2:**
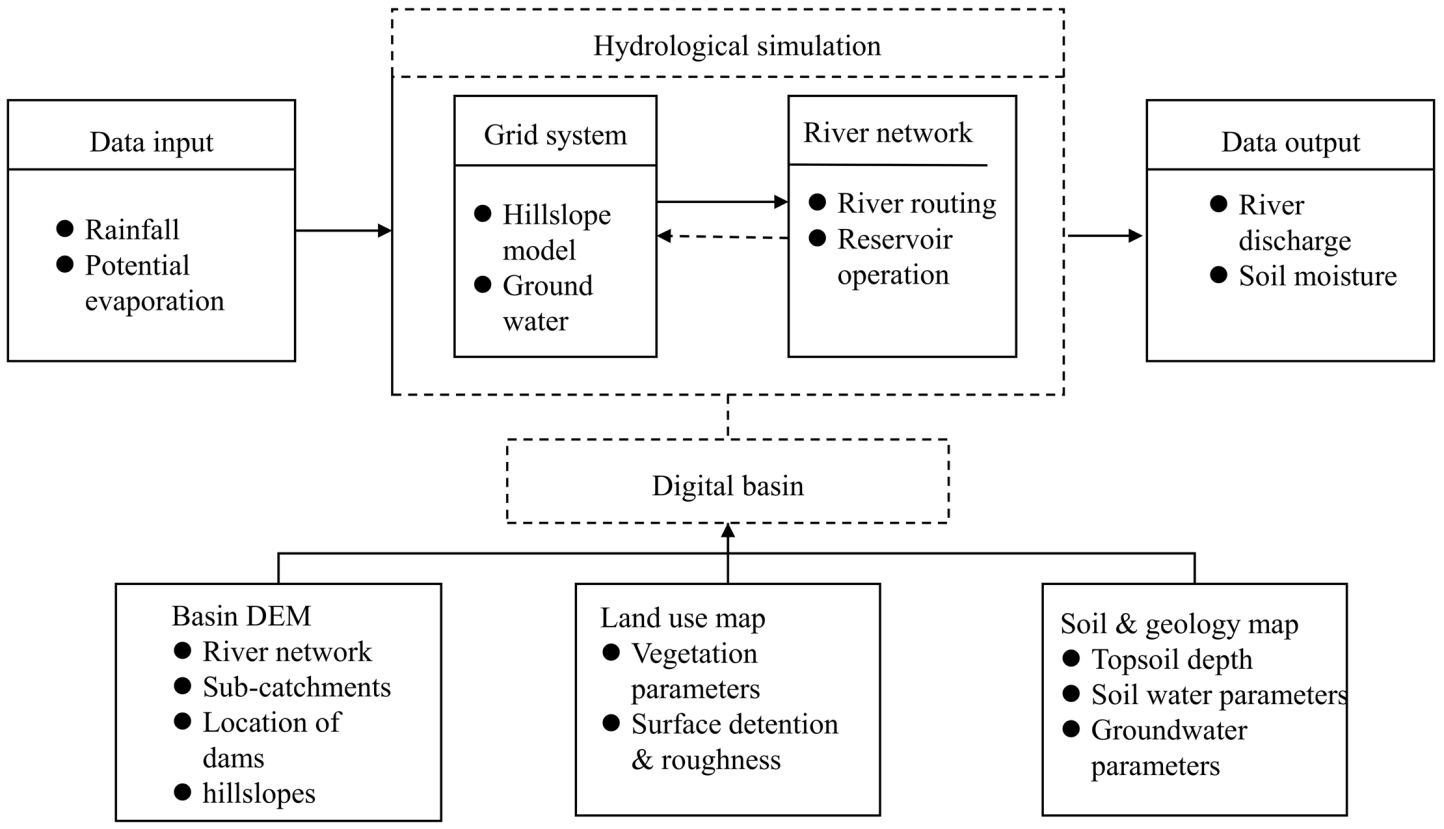
GBHM structure.

The GBHM is used in this study [24]. The model consists of four parts, as shown in Fig 2. The GBHM has been successfully applied to many different types of rivers, ranging from the catchment scale [25–27] to the continental scale [14]. The model has also been used to reliably forecast floods [28], assess the impacts of land use change [29], evaluate the effects of climate change [30], support dam operations [31] and improve flood management [27].

The key characteristics of the GBHM include a flow-interval hill slope discretization scheme, a kinematic wave for river network routing and the ability to simulate physically physically based hydrology on a hill-slope. The digital basin is defined using a digital elevation model (DEM) [32]. The digital basin is subdivided into a number of cascade-connected flow intervals following the flow distance from outlet to upper source using the area and width functions to lump the topography and divide the catchments into a series of flow interval hillslopes [33]. The hillslope is the fundamental computation unit in the model, providing lateral inflow estimates to the same portion of the main stream (see [24] for further details). The catchment parameters related to topography, land use and soil are then calculated for each simulation unit. By establishing a digital basin, the study basin can be divided into a discrete grid system. The grid is represented by a number of geometrically symmetrical hill slopes. The complex, two-dimensional water kinematics can be simplified to a single dimension by applying this flow-interval and hillslope-river based scheme of sub-grid parameterization. A physically based model is used to simulate the hydrological processes of snowmelt, canopy interception, evapotranspiration, infiltration, surface flow, subsurface flow and the exchange between the groundwater and the river for each hillslope. Finally, a nonlinear, numerical river routing scheme is used to calculate the catchment runoff [33].

According to a report from the Mekong River Commission (MRC), only 10 per cent of the estimated hydroelectric potential of the Lower Mekong Basin has been developed at present. Additionally, no dams exist on the main channel in the Lower Mekong Basin [34]. The Upper Mekong, or Lancang Jiang, is projected to have a total active reservoir storage that is 30% of the annual mean discharge in 2020. Lancang Jiang only contributes 16% of the total basin runoff [1]. Dams in China only impact 4.8% of the total runoff; although, they may considerably impact the Upper Mekong. Because the Mekong’s flow has not yet been drastically modified by human development, especially in the LMB, dam operations are neglected in this study. Thus, only natural hydrological processes are included, and reservoir controls are ignored, despite the fact that the GBHM possesses associated functions. Forty-nine sub-catchments are distinguished in the study using the Pfafstetter basin numbering system [35].

### Data

The geographical data used in this study were obtained from a number of global public data sets. The 5 km×5 km computational grid was extracted and resampled from 90 m resolution digital elevation model (DEM) data provided by the USGS (available from http://globalchange.nsdc.cn/page/index.vpage). The land use and land cover data were downloaded from the USGS 1-km Global Land Cover Characteristics Database version 2.0 [36]. Soil type and soil depth data were obtained from the Food and Agriculture Organization (FAO) digital soil map of the world and the associated soil properties.

In situ observation data for the LMB was obtained from the MRC historical observation data set. This data set includes air temperature (mean, max and min), discharge, precipitation, relative humidity, sunshine duration and wind speed. The same meteorological elements were collected for the Upper Mekong from the China surface climate dataset (Daily), which is produced by the China Meteorological Data Sharing Service System (CMDSS). As shown in Fig 1(B), we collected data from 321 rainfall stations and 102 meteorological stations in the MRB (see S1 File for the detail gauge meteorological data in MRB); however, the entire time series from 1998 to 2012 was only available for a few gauge stations. Among these stations, the rain gauge station and meteorological station data provided by CMDSS are of higher quality and encompass full time series with even distributions; however, only 12 gauges are available. Numerous MRC stations are available, but the data are sporadic with uneven distributions, which may impact the simulations. Discharge time series were obtained from six MRC stations on the main stream, namely Chiang Saen, Luang Prabang, Nong Khai, Mukdahan, Pakse and Stung Treng from upstream to downstream. The control area and runoff information are listed in Table 1. In the Mekong Delta the main stream breaks into nine branches that discharge into the ocean. The Stung Treng station controls more than 90% of the whole of the Mekong’s discharge; therefore, our simulations were only conducted above this station.

**Table 1:** Discharge station information.

Station	Drainage area 10^4^ km^2(ratio,%)	Average runoff m3/s (ratio,%)
Chiang Sean	18.9(23.8)	2688(18.6)
Luang Prabang	26.8(33.7)	3913(27.0)
Nong Khai	30.2(39.7)	4422(30.3)
Mukdahan	39.1(49.2)	7782(53.7)
Pakse	54.5(68.6)	9880(68.2)
Stung Treng	63.5(79.9)	13133(90.1)

The Tropical Rainfall Measuring Mission Multi-satellite Precipitation Analysis Product 3B42 Research Version 7 (Abbreviated to TRMM 3B42V7 in this paper) daily precipitation data were used as remotely sensed rainfall in this study (see S1 File for extracted precipitation data form TRMM 3B42V7 in MRB). This data set was originally developed by the National Aeronautics and Space Administration (NASA) at fine spatial and temporal resolutions (0.25° × 0.25° and 3-hourly). The data encompass areas between 50°N and 50°S from 1998 to 2012, which is when version 7 was released. The data set was calibrated and merged with rain gauge observations provided by the Global Precipitation Climatology Centre (GPCC; GPCP global monthly rain gauge analysis) to remove the satellite retrieval bias at a monthly scale [37]. Our model utilizes a 5 km×5 km computational grid; therefore, these two rainfall data sets were interpolated to the correct grid size. An angular distance weighting method (detailed description provided in [38]) was applied to generate a rainfall field. The evapotranspiration in the model was calculated using meteorological station data via the Penman-Monteith equation recommended by the FAO. These data were then interpolated to the computational grid using the same method.

### Assessment indices

Three indices were used to measure the model performance for each numerical experiment: the ratio of the absolute error to the mean (*RE*), the modified Nash-Sutcliffe coefficient (*NASH*) and the Root-mean-square error (*RMSE*). The indices were used to evaluate the agreement between the simulated and observed hydrographs at different temporal scales. The following equations were used in this study, in which $\bar{obs}$ and $\bar{sim}$ represent the mean observed and simulated discharges, respectively, and subscript *i* refers to the time (day or month). A smaller *RE* or *RMSE* indicates a better simulated discharge result. *NASH* ranges from negative infinity to 1. The closer to 1, the better the simulation of the discharge hydrograph [39].

$$RE=\frac{\bar{sim}-\bar{obs}}{\bar{obs}}\times 100\%$$ (1)

$$NASH=1-\frac{\sum_{i=1}^{T}{\left({obs}_{i}-{sim}_{i}\right)}^{2}}{\sum_{i=1}^{T}{\left({obs}_{i}-\bar{obs}\right)}^{2}}$$ (2)

$$RMSE=\sqrt{\frac{\sum_{i=1}^{n}{\left({obs}_{i}-{sim}_{i}\right)}^{2}}{n-1}}$$ (3)

## Results

### Results of the GBHM driven by in situ rainfall

**Fig 3:**
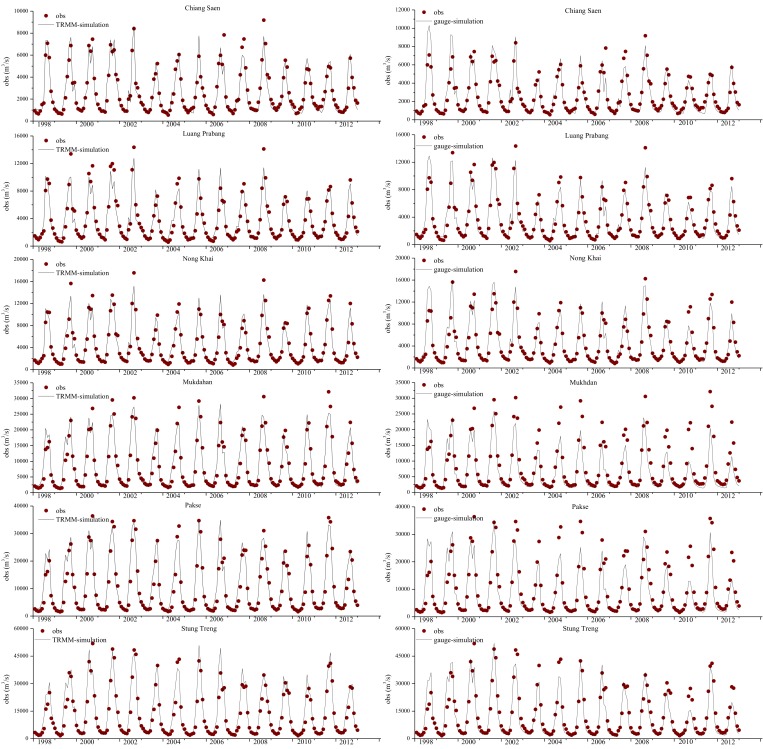
Comparison of simulated and observed discharge values at six main stream stations: the left ones are results of the model driven by TRMM3B42V7 data while the right ones are results of the model driven by gauge rainfall data.

First in situ rainfall was used as the driver for the model. It was calibrated against daily discharge data observed at the six stations from 1998 to 2001. We adjusted bio-physical parameters to improve the simulation precision of the river discharge. After calibration, the model performed well, with *NASH* values higher than 0.6 and *RE* values less than 10% at the majority of the six stations, as listed in Table 2. We also found that the simulated discharge was less accurate at upstream stations, especially at Chiang Saen. These inaccuracies are likely caused by dam operations. The GBHM did not include a reservoir module in this study. However, these results suggest that dam operations impacted the upstream discharge processes. After calibration, the daily discharge was simulated for validation from 2002 to 2012. The results were compared with the observation data to evaluate the model’s effectiveness. The results for six stations are shown in Fig 3(A). The daily-scale *NASH* and *RE* values are listed in Table 2.

**Table 2:** Discharge simulation results of two calibration and validation experiments: *RE*, *NASH* and *RMSE* values (m^3^/s).

Station	Calibration(1998–2001)			Validation(2002–2012)		
	RE(%)	NASH	RMSE	RE(%)	NASH	RMSE
Gauge-Chiang Saen	8.3	0.508	1679	-19.8	0.588	1190
Gauge-Luang Prabang	-1.9	0.722	2216	-16.1	0.655	1557
Gauge-Nong Khai	7.5	0.620	2860	-12.8	0.621	2036
Gauge-Mukdahan	-9.2	0.688	4995	-17.2	0.693	3927
Gauge-Pakse	-2.8	0.670	6479	-20.1	0.669	4917
Gauge-Stung Treng	-2.6	0.722	8419	-15.8	0.741	5536
TRMM 3B42 V7-Chiang Saen	2.3	0.656	1402	-7.5	0.644	1106
TRMM 3B42 V7-Luang Prabang	-8.5	0.684	2250	-0.8	0.679	1504
TRMM 3B42 V7-Nong Khai	2.1	0.679	2418	-1.2	0.673	1890
TRMM 3B42 V7-Mukdahan	12.8	0.642	4853	-1.0	0.747	3565
TRMM 3B42 V7-Pakse	12.6	0.675	5870	6.2	0.738	4375
TRMM 3B42 V7-Stung Treng	9.7	0.724	7617	10.2	0.754	5398

Table 2 shows that the model performs well at a daily scale during the validation period, with *RE* values less than 20% and *NASH* values greater than 0.6 at all six stations except Chiang Saen. *NASH* values at the upper stations (Chiang Saen, Luang Prabang and Nong Khai) were generally smaller than those at the lower stations (Mukdahan, Pakse and Stung Treng). This trend is due to the dam’s impact, as previously described. Note that *RE* is always negative during the validation period, implying that the simulated discharge is consistently lower than the observed discharge. Compared with the model’s performance during the calibration period, we speculate that either the parameters that were calibrated for 1998–2001 or the forcing data (or both) are problematic. Fig 3 illustrates that year 1998–1999 is a dry period in the Mekong compared to other years, especially at the lower stations. The extremely dry period during the calibration period may have caused the optimal parameters for dry years to underestimate the discharge in wet years. As shown in Fig 1(B), the rain gauges are unevenly distributed. Some areas have multiple gauges while other areas have none. The MRB climate is complex and highly heterogeneous; therefore, such an uneven rain gauge distribution may impact the interpolations. The sparsely distributed station data may not be suited for a spatially distributed hydrological model in this region. Spatially distributed forcing data, such as remote sensing products or reanalysis data, may provide a suitable alternative.

### Results of GBHM driven by TRMM 3B42V7

To assess the reliability of the station data, the forcing rainfall data used in the previous section was replaced with the TRMM 3B42V7 rainfall. The model was also calibrated for the period of 1998–2001 and validated for 2002–2012.

Fig 3(B) presents a comparison of the observed daily discharges generated using the gauge data and TRMM 3B42V7 data after calibration. Table 2 shows that the *NASH* and *RE* results for this scenario are similar to the results from the scenario driven by in situ data; although, the TRMM simulation slightly overestimates discharge at the lower stations (Mukdahan, Pakse and Stung Treng). The *RMSE* results suggest that the simulated discharge more precisely reflects the observations when using the TRMM 3B42V7 data. The TRMM simulation also obtained a poor result at Chiang Saen; however, it did improve upon the gauge simulation.

During the validation period, the model performance was stable and compared well to observations, with *RE* values of less than 10% and *NASH* values higher than 0.6 at all six stations. Comparing the validation results of these two experiments, the model driven by TRMM 3B42V7 gives better results at all stations based on *RE*, *RMSE* and *NASH* values. In addition, the decreasing trend in the simulated discharge generated using the model driven by TRMM 3B42V7 is similar to that produced by the in situ model during the validation period. The *RE* values in four of the six stations tended to “decrease” (from positive to negative at Chiang Saen, Nong Khai and Mukdahan, or from positive to less positive at Pakse), but much less so compared to the gauge simulation. This indicates that the optimal parameters for dry years are not suitable for wet years; however, this impact is small compared to the influence observed in the gauge simulation. Such results may imply that TRMM 3B42V7 rainfall data are more suitable for driving a distributed hydrological model than are in situ data.

**Fig 4:**
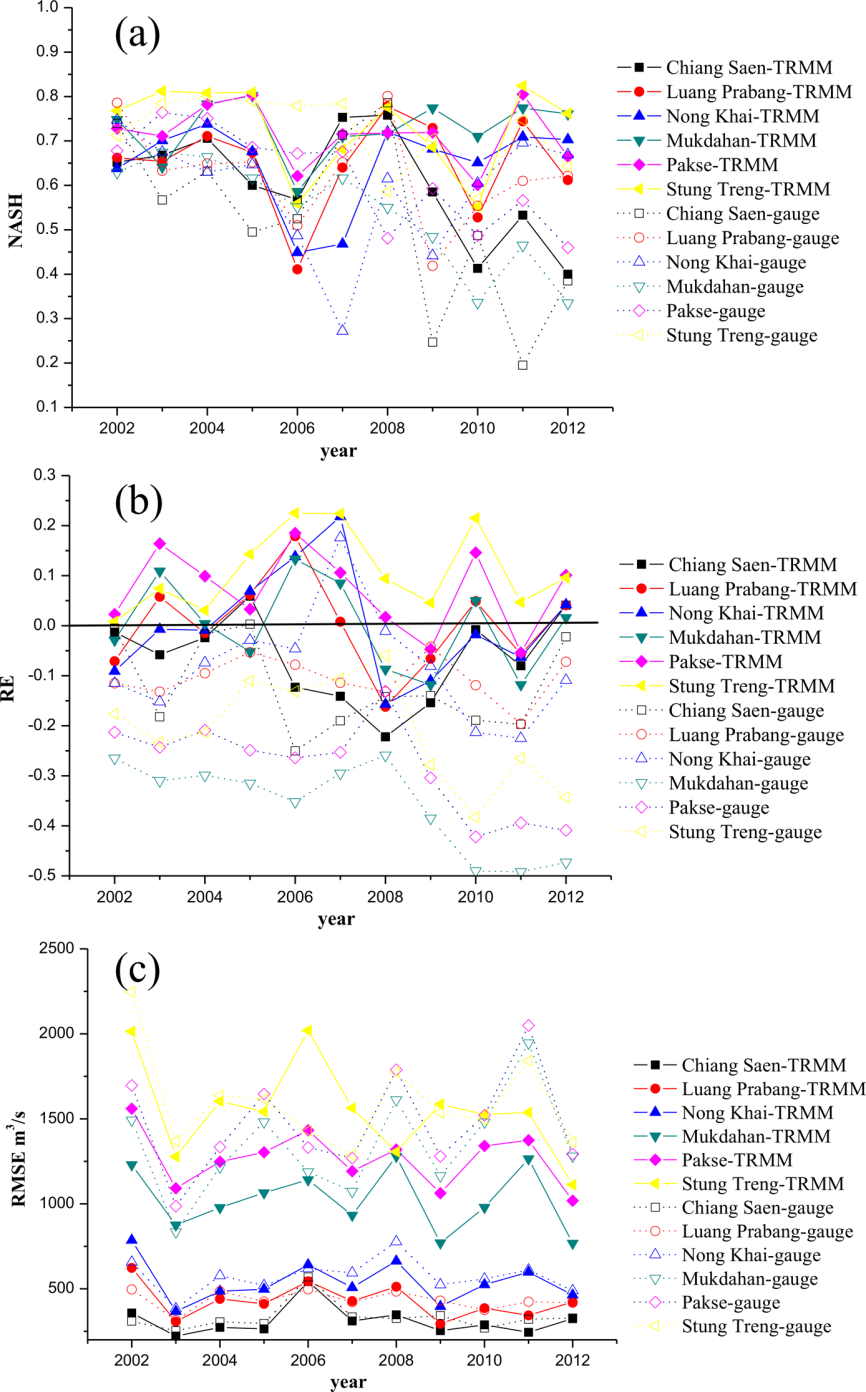
*NASH* (a), *RE* (b) and *RMSE* (c) values from two simulated discharge experiments for each year during 2002–2012: the solid lines represent the TRMM simulation while the dashed lines represent the gauge simulation.

To compare these two experiments further, the *NASH*, *RE* and *RMSE* values for each year were calculated for the two experiments, as shown in Fig 4.

For daily *NASH*, Fig 4 illustrates that the solid lines, which represent the results of the TRMM simulation, are generally higher than the dashed lines, which represent the results of the gauge simulation. Additionally, the solid lines are more stable at a high level, while the dashed lines are more erratic. The *RE* values in each year are similar for both experiments. The simulated discharge from the model driven by TRMM 3B42V7 is much closer to the observations than are the discharge results from the in situ-driven simulation. The gauge simulation provides a clear underestimation for the entire period and for both stations. During the later years, both simulations underestimate the discharge; although, the error for the TRMM 3B42V7 driven model is smaller. This may be caused by the parameters, which were calibrated in a very dry year, as explained in the previous section. Similarly, the *RMSE* values for the TRMM simulation are generally much smaller than the *RMSEs* of the gauge simulation.

**Fig 5:**
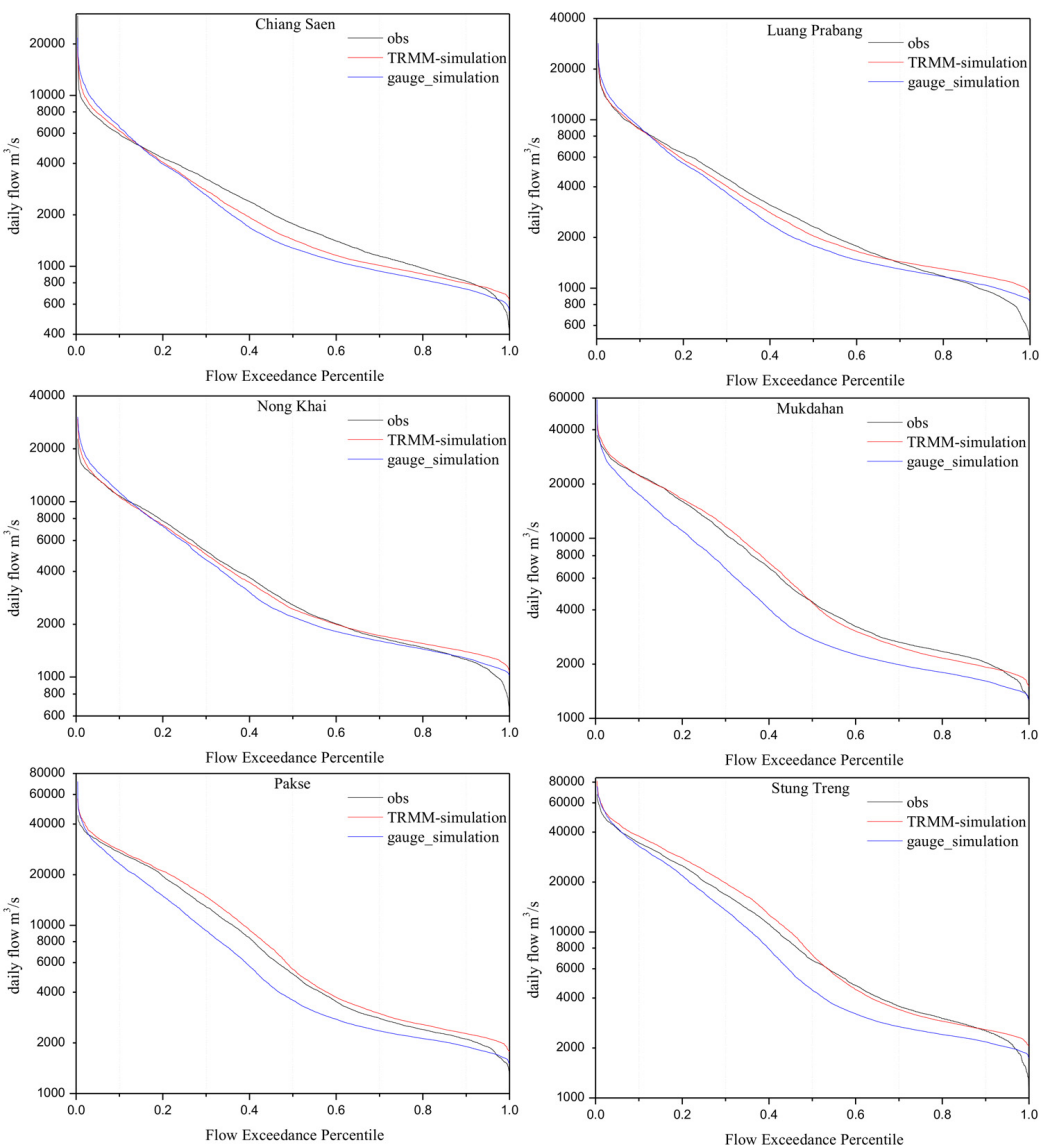
Daily discharge Flow Duration Curves for observation, TRMM simulation and gauge simulation data at six main stream stations from 1998 to 2012.

In addition, Fig 5 illustrates the Flow Duration Curves (FDC) for the two simulations at a daily scale from 1998 to 2012 at the six stations. Per Fig 5, the gauge simulation performs well at the three upper stations (Chiang Saen, Luang Prabang and Nong Khai). Conversely, the FDCs of the gauge simulation are apparently lower than the observations at Mukdahan, Pakse and Stung Treng. However, the FDCs produced by the TRMM simulation consistently agree with the observations. Therefore, the TRMM simulation performs better than the gauge simulation on the basis of FDCs. This result further proves TRMM 3B42V7’s ability and advantages for use in distributed hydrological models, such as the GBHM.

## Discussion

The length of the calibration period was changed to further test the stabilities of the gauge simulation and the TRMM simulation. We used periods of 2 years (1998–1999), 4 years (1998–2001) and 8 years (1998–2005) to calibrate the GBHM. The *RE*, *NASH* and *RMSE* values were then calculated for the validation periods, namely 2000–2012 for the 2 year calibration, 2002–2012 for the 4 years calibration and 2006–2012 for the 8 year calibration. The *RE* and *NASH* values for the calibration and validation periods of the two simulations are listed in Table 3 and Table 4 for these three scenarios. The simulated discharge results can be found in S1 File. As shown in Table 3, a longer calibration period generally leads to a more consistent *RE*, suggesting that the model is more stable. Moreover, the *RE* values of the TRMM simulation generally change less than those of the gauge simulation during the validation period. This indicates that the TRMM simulation is less sensitive to the length of the calibration period compared to the gauge simulation. In contrast to *RE*, the *NASH* behavior varies for the 2 year, 4 year and 8 year calibrations. To some extent, *NASH* variation patterns can be observed based on the calibration period. However, in most cases, the TRMM simulation *NASH* values decrease less (or increase more) than the gauge simulation *NASH* values. This result further indicates that the TRMM simulation is more stable than the gauge simulation.

**Table 3:** *RE* values at each station for 2 year, 4 year and 8 year calibration periods for the TRMM simulation and gauge simulation. Bold numbers represent a better result compared to the other simulation (i.e., less variation).

Scenarios	TRMM-simulation			Gauge-simulation		
	Calibration(%)	Validation(%)	Change^*^	Calibration(%)	Validation(%)	Change
Chiang Saen-2 years	1.2	-13.4	**0.15**	-3.9	-19.9	0.16
Chiang Saen-4 years	2.3	-7.5	**0.10**	8.3	-19.8	0.28
Chiang Saen-8 years	0.1	-11.0	**0.11**	-7.0	-22.9	0.16
Luang Prabang-2 years	-3.9	-8.5	**0.05**	-7.7	-16.5	0.09
Luang Prabang-4 years	-8.5	-0.8	**0.08**	-1.9	-16.1	0.14
Luang Prabang-8 years	-4.4	-1.6	**0.03**	-11.9	-16.0	0.04
Nong Khai-2 years	0.9	-4.9	**0.06**	5.2	-10.6	0.16
Nong Khai-4 years	2.1	-1.2	**0.03**	7.5	-12.8	0.20
Nong Khai-8 years	-1.4	-3.8	**0.02**	-4.1	-11.9	0.08
Mukdahan-2 years	-6.1	-26.6	**0.21**	-2.8	-34.2	0.31
Mukdahan-4 years	12.8	-1.0	**0.14**	-9.2	-17.2	0.08
Mukdahan-8 years	5.3	-2.2	0.08	3.5	-20.8	**0.24**
Pakse-2 years	1.1	-16.0	**0.17**	-8.3	-34.6	0.26
Pakse-4 years	12.6	6.2	**0.06**	-2.8	-20.1	0.17
Pakse-8 years	9.8	5.8	**0.04**	-2.6	-22.7	0.20
Stung Treng-2 years	7.0	-8.6	**0.16**	-5.0	-25.9	0.21
Stung Treng-4 years	9.8	10.2	**0.00**	-2.7	-15.8	0.13
Stung Treng-8 years	7.9	13.1	**0.05**	-5.3	-16.7	0.11

* Change is calculated as the absolute value of RE(in validation)-RE(in calibration). It indicates how much RE change in validation period.

**Table 4:** *NASH* values at each station for 2 year, 4 year and 8 year calibration periods for the TRMM simulation and gauge simulation. Bold numbers represent a better result compared to the other simulation (i.e., decrease less or increase more).

Scenarios	TRMM-simulation			Gauge-simulation		
	Calibration	Validation	Change^*^	Calibration	Validation	Change
Chiang Saen-2 years	0.737	0.651	-0.086	0.584	0.579	**-0.005**
Chiang Saen-4 years	0.656	0.644	-0.012	0.508	0.588	**0.080**
Chiang Saen-8 years	0.671	0.628	-0.043	0.593	0.574	**-0.019**
Luang Prabang-2 years	0.689	0.694	**0.005**	0.705	0.654	-0.051
Luang Prabang-4 years	0.684	0.679	**-0.006**	0.722	0.655	-0.067
Luang Prabang-8 years	0.694	0.675	**-0.019**	0.700	0.646	-0.054
Nong Khai-2 years	0.684	0.692	**0.009**	0.634	0.614	-0.019
Nong Khai-4 years	0.679	0.673	-0.006	0.620	0.621	**0.001**
Nong Khai-8 years	0.698	0.676	**-0.022**	0.652	0.592	-0.060
Mukdahan-2 years	0.736	0.678	**-0.059**	0.628	0.558	-0.070
Mukdahan-4 years	0.642	0.747	**0.105**	0.688	0.693	0.005
Mukdahan-8 years	0.714	0.737	0.022	0.651	0.680	**0.029**
Pakse-2 years	0.760	0.769	**0.008**	0.626	0.560	-0.066
Pakse-4 years	0.675	0.738	**0.063**	0.669	0.669	0.000
Pakse-8 years	0.729	0.721	**-0.008**	0.681	0.626	-0.055
Stung Treng-2 years	0.770	0.818	**0.048**	0.715	0.720	0.005
Stung Treng-4 years	0.724	0.754	**0.029**	0.722	0.741	0.019
Stung Treng-8 years	0.766	0.712	-0.055	0.736	0.717	**-0.019**

* change is calculated as NASH in validation period minus NASH in calibration period.

Therefore, it is clear that the GBHM driven by TRMM 3B42V7 performed much better during the validation period than did the model driven by the in situ data, indicating that the model driven by TRMM 3B42V7 is much more stable and reliable.

**Fig 6:**
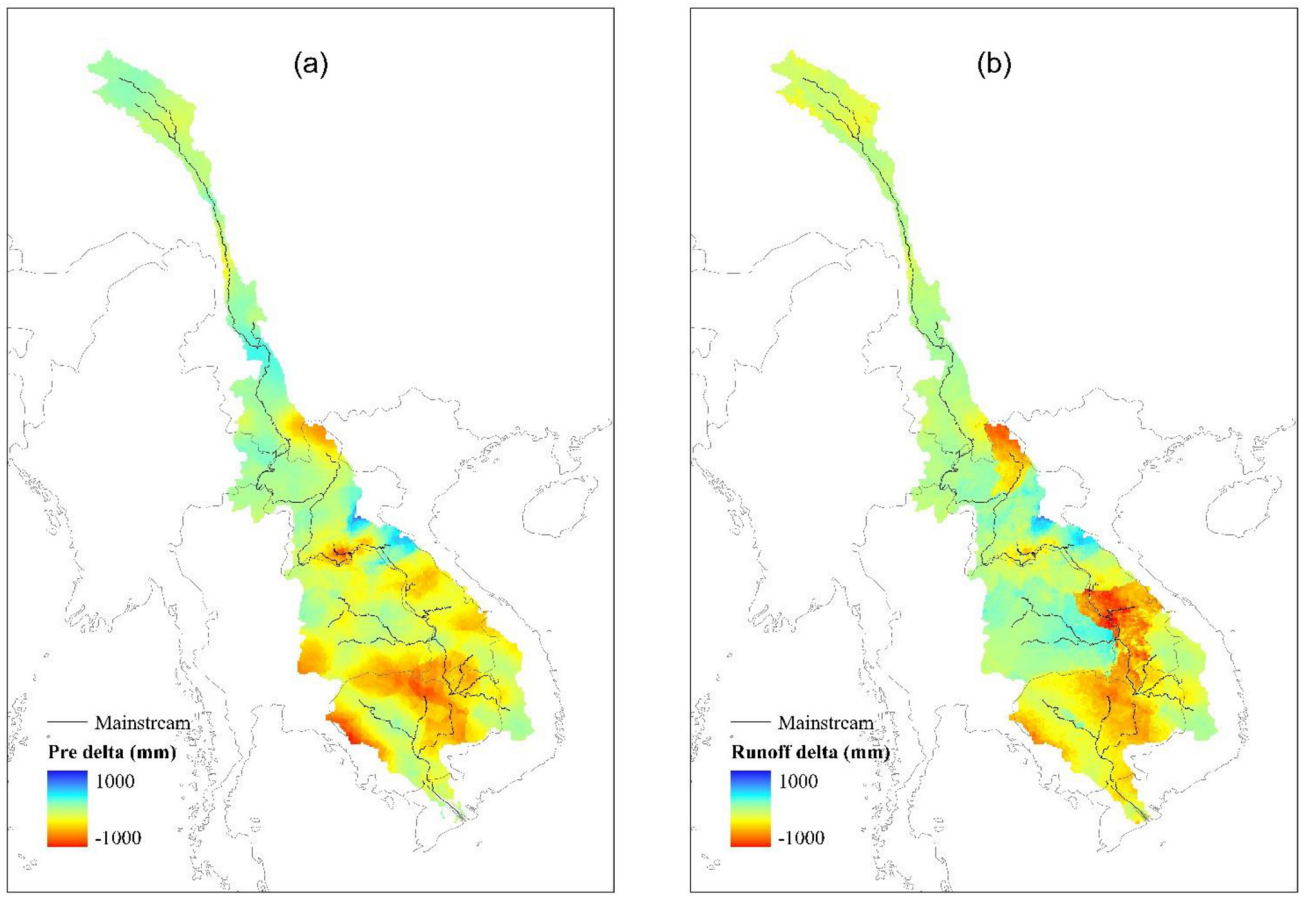
Input rainfall field (a) and generated runoff (b) difference between the two experiments.

The year 2003 is used as an example for further analysis. From Figs 3 and 6, it is clear that, the discharge simulated by the TRMM 3B42V7 is much closer to the observed discharge than the discharge simulated by the gauge simulation. The gauge simulation shows an obvious underestimation for the entire period and for both stations, while the TRMM simulation provides a slight overestimation at several stations. Furthermore, runoff values generated by each experiment are compared for each grid in Fig 6(B). Fig 6(B) shows that the generated runoff values driven by in situ data are underestimated by 239 mm with respect to the TRMM 3B42V7 driven simulation, especially in the left bank of the lower basin, which contributes the majority of the basin’s discharge. Fig 6(A) illustrates that the underestimation is due to the input rainfall bias, as the 2003 the average rainfall in the basin was 1441 mm when interpolated using gauge station rain data and 1263 mm when interpolated using the TRMM 3B42V7, resulting in a 178 mm positive bias. The patterns illustrated in Fig 6(A) and Fig 6(B) are similar. We can conclude that the simulated discharge differences in the former section are mainly caused by input rainfall field differences.

We must determine what causes the differences between the input rainfall fields of the two experiments. Noting the uneven spatial distribution of rain gauge stations in Fig 2, we believe that the uneven distribution of gauge stations causes the errors associated with the input rainfall field.

**Fig 7:**
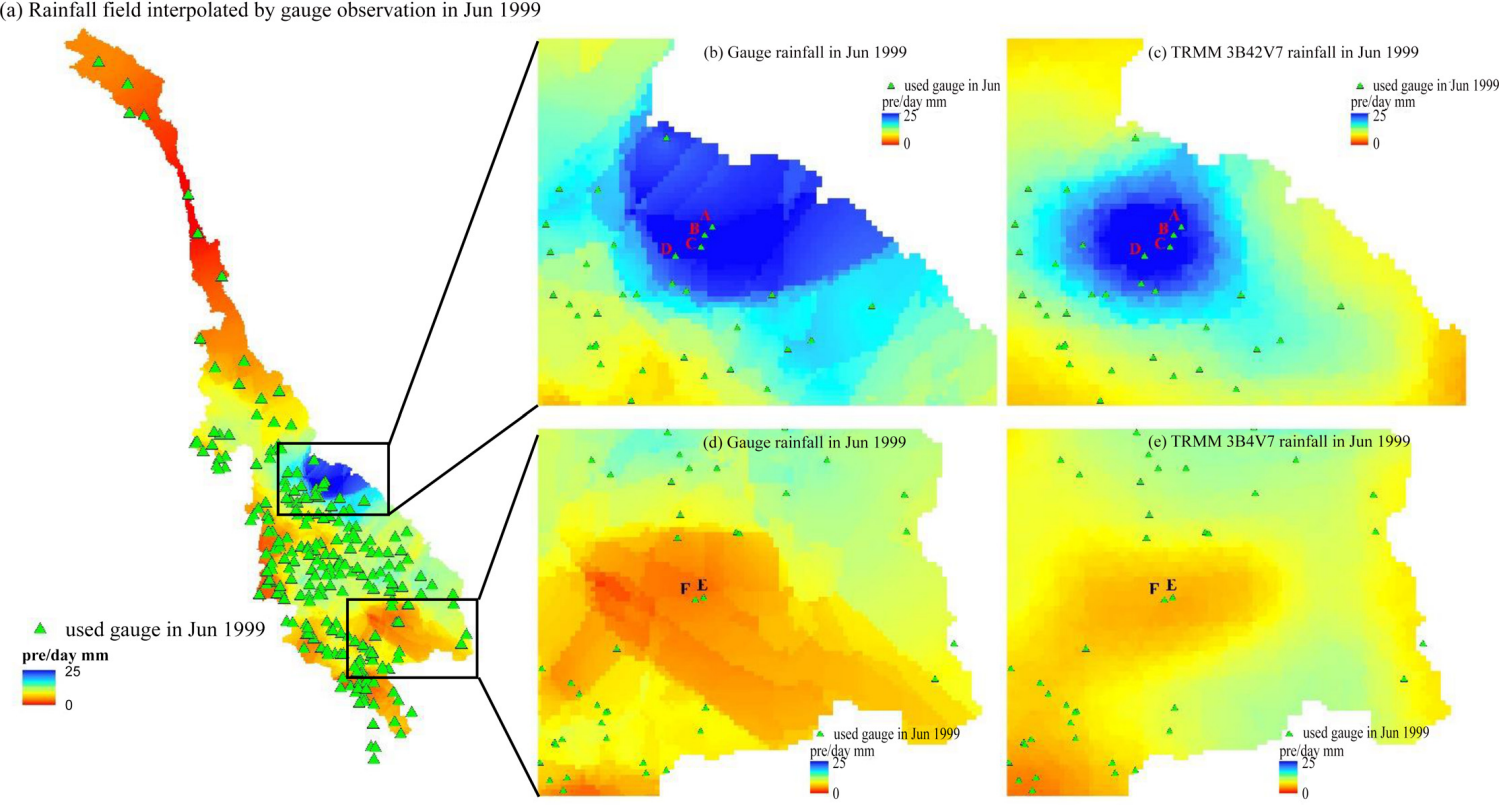
Rainfall field from June 1999: (a) rainfall field interpolated using gauge observations and the gauges used in June 1999; (b) and (d) provide magnified images of the two black rectangles in (a); (c) and (e) show the rainfall fields for the same two rectangles interpolated from TRMM 3B42V7 data.

A case study was then chosen for further analysis. We studied the two areas indicated by the rectangles in Fig 7(A), which illustrates the rainfall field based on the gauge observations. Fig 7(B) and 7(D) show the magnified image extracts from the two rectangles, while Fig 7(C) and 7(E) show the rainfall fields for the same two rectangles interpolated using TRMM 3B42V7 data. In these two areas, the TRMM 3B42V7 rainfall field is significantly different than the gauge interpolation result. Because RS data can effectively represent spatial patterns, we can assume that the true rainfall field is more or less equivalent to the pattern shown in Fig 7(C) and 7(E), ignoring some inherent bias. Fig 7(C) shows that gauges A, B, C and D are located in the center of the rainfall field. However, no gauge observations are available in the relatively low rainfall region to the east of this area where rainfall is relatively low. Thus, the GBHM use gauges A, B, C and D to estimate the rainfall rate to the east of this area, which cause an overestimation compared to the real rainfall field. A similar phenomenon can be observed in Fig 7(D) and 7(E), where gauges E and F lead to an underestimation.

Therefore, the uneven distribution of rain gauges causes the gauge simulations to be more sensitive to the length of the calibration period or the choice of calibration period (i.e. a dry year or wet year). During a dry year, the gauge observation may be much lower. If the gauge distribution is uneven, then dry trends may be intensified via interpolation. Similarly, wetter rainfall fields may be interpolated during wet year. The uneven distribution of rainfall gauges causes the gauge rainfall field to be more heterogeneous than the TRMM 3B42V7 based rainfall field, making the gauge simulation more sensitive to dry or wet years.

**Fig 8:**
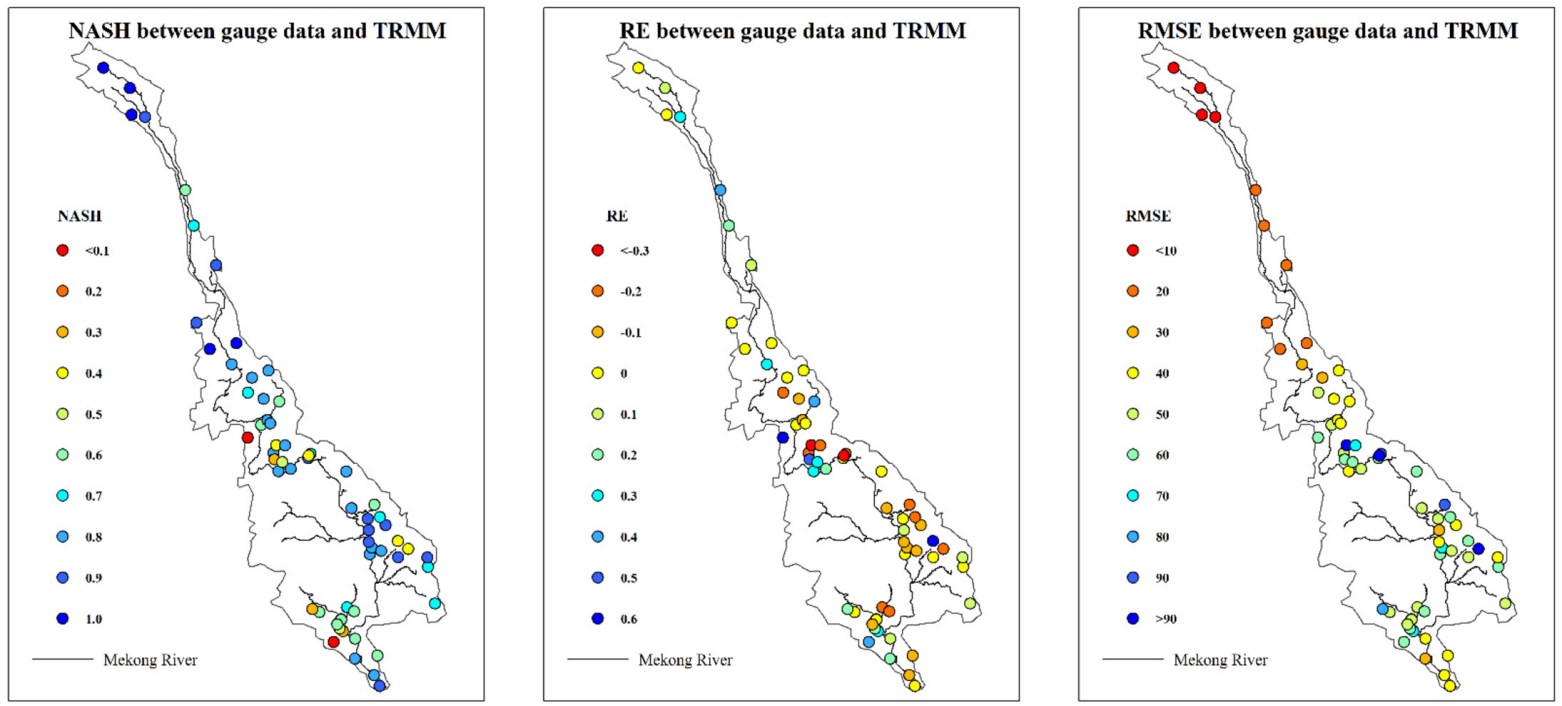
Pixel-point comparison between gauge data and TRMM 3B42V7 data at the 62 gauges.

**Fig 9:**
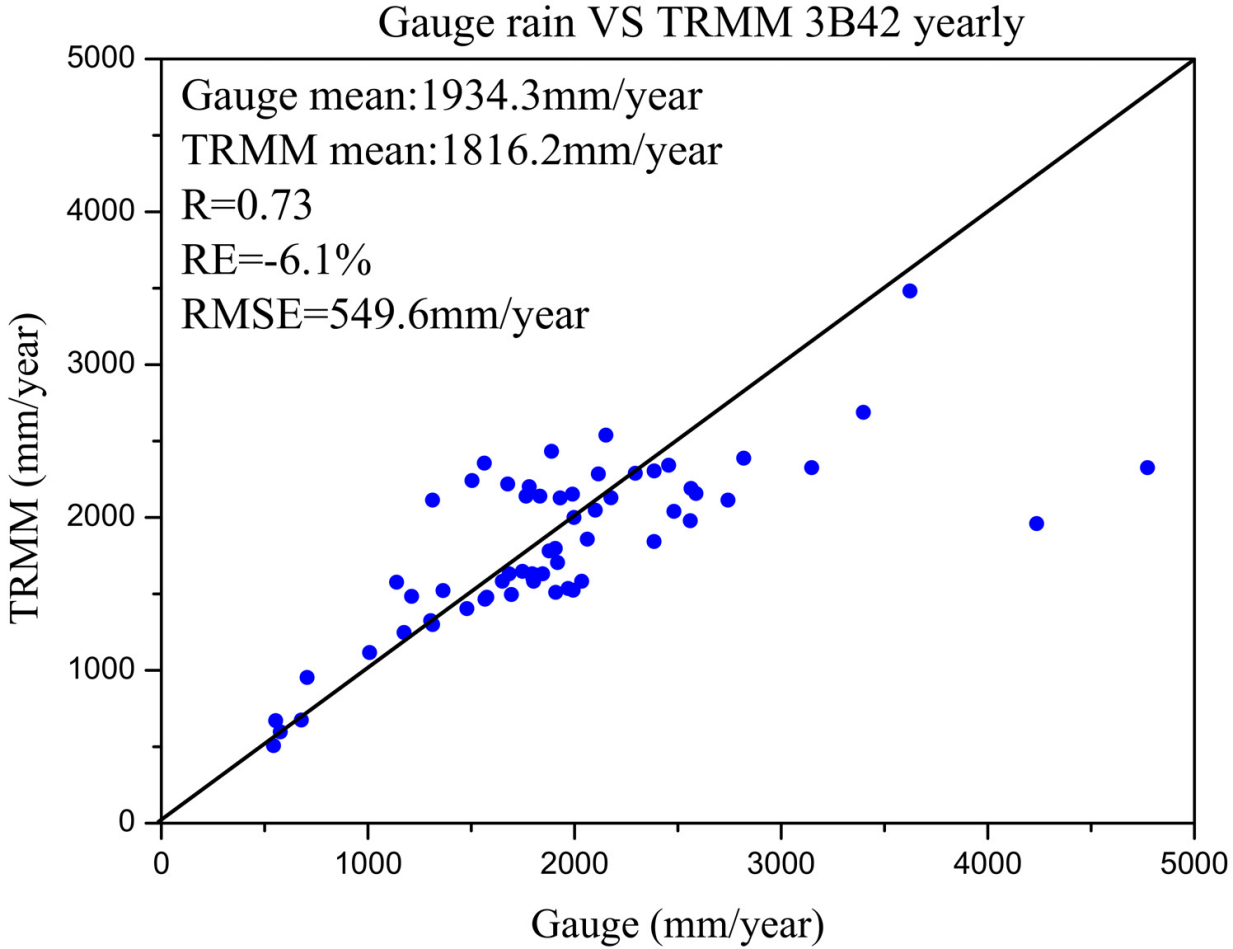
Comparison of yearly rainfall between gauge rain data and TRMM 3B42V7 at the 62 stations.

To further test this hypothesis, 62 rainfall gauges with a full time series from 1998 to 2004 were selected to compare with the TRMM 3B42V7 data set. To avoid interpolation errors, we directly conducted a pixel–point comparison at the 62 gauges. Three statistical indices (*RMSE*, *RE* and *NASH*) were calculated to estimate the differences between these two rainfall data sets at a monthly scale. Fig 8 shows the results. Excluding a few gauges (mainly in the lower basin), the rain gauge data are consistently mimicked by the TRMM 3B42V7 data. Fig 9 demonstrates this trend more directly using a scatter diagram of average yearly rainfall between gauge data and TRMM 3B42V7 data. Except for two points, the remainder of the stations exhibit good agreement with the TRMM 3B42V7 estimation. This result may imply that the rain gauge data are reasonably consistent with the TRMM 3B42V7 data at the local scale. Moreover, we can also see that no gauge stations are present (Fig 8) in the area where the rainfall field was underestimated by in situ data lower (Fig 6(A)). Therefore, the rainfall data interpolated by a distant gauge are not reliable in areas with few or no gauges. However, TRMM 3B42V7 is grid based and spatially distributed, which makes the calculated rainfall field closer to the real rainfall field. Thus we conclude that the uneven distribution of gauge stations causes the errors associated with interpolating the rainfall field, which ultimately causes the errors observed in the simulated discharge results.

## Conclusions

This study demonstrates the development of a remote sensing-based distributed hydrological model (i.e. GBHM), in which the fundamental data, parameters and forcing data are all provided via RS. The results demonstrate that such a distributed hydrological model can effectively simulate hydrological processes in the MRB. The model was driven with both in situ rainfall data and TRMM 3B42V7 data to assess the feasibility of simulating hydrological processes in a poorly gauged basin using RS rainfall data (i.e. TRMM 3B42V7).

A detailed comparison was conducted between the TRMM simulation and the gauge simulation based on different scales and variations. The discharge produced when TRMM 3B42V7 was used to drive the GBHM is much closer to the observations compared to the discharge generated when unevenly distributed gauge data were used to drive the model. The FDCs produced by the TRMM simulation are more similar to the observed FDCs. Moreover, the calibration period was varied, proving that the gauge simulation is more sensitive to the calibration period, while the TRMM simulation does not heavily depend on the length of the calibration period. Further analysis reveals that the uneven distribution of rain gauges causes the input rainfall field to be less representative and more heterogeneous, which worsens the simulated discharge results. This trend explains why the gauge simulation is more sensitive to dry years, wet years and the choice of the calibration period. On the contrary, TRMM 3B42V7 is grid based and evenly distributed. It can effectively represent spatial patterns of rainfall and provide a more stable rainfall field than that produced using sparsely distributed gauge observations.

These experiments demonstrate that the parameters used in DHMs significantly impact the results. The effectiveness of parameters depends heavily on the characteristics of the input data (both spatial and temporal characteristics). Poor representative input data (such as using unevenly distributed gauge data or a very dry/wet period as the calibration period) may cause the optimized parameters from calibration period to be ineffective during the validation period. Our work shows that parameters calibrated using a remote sensing rainfall product are more stable and effective than those calibrated using gauge data. The impact of choosing a dry/wet year for calibration was much smaller for the results driven by RS rainfall data compared to those driven by gauge data, as grid-based RS rainfall data are less sensitive to the choice of the calibration period.

Distributed hydrological model are significantly impacted by different input sources, especially rainfall sources, which influence the model parameters and simulation results. Sparsely distributed gauge data may be less representative and problematic, while RS data are able to drive the DHM and provide more reliable hydrologic predictions in this region, as RS data are spatially distributed. From this perspective, RS rainfall data may be more suitable for use in distributed hydrological models, especially in basins with poor or ungauged data. RS-based DHMs provide an effective alternative in ungauged basins, and may provide better solutions than conventional methods in some cases.

Some limitations exist in this study. Although our study shows that TRMM 3B42V7 provides a better input rainfall product than the unevenly distributed gauge observations for the GBHM, this trend should be tested in other cases, such as using different RS-based rainfall data for different DHMs in different basins. Both gauge observation and RS-based rainfall data have advantages and disadvantages. Effectively combining these advantages and avoiding disadvantages requires further study.

## Supporting Information

S1 File Gauge rainfall data, TRMM 3B42V7 rainfall data and simulated results of the two experiments. (RAR)
